# Unifying Speed-Accuracy Trade-Off and Cost-Benefit Trade-Off in Human Reaching Movements

**DOI:** 10.3389/fnhum.2017.00615

**Published:** 2017-12-19

**Authors:** Luka Peternel, Olivier Sigaud, Jan Babič

**Affiliations:** ^1^HRII Lab, Advanced Robotics, Istituto Italiano di Technologia, Genoa, Italy; ^2^Department for Automation, Biocybernetics and Robotics, Jožef Stefan Institute, Ljubljana, Slovenia; ^3^Sorbonne Universités, UPMC Univ Paris 06, CNRS UMR 7222, Institut des Systèmes Intelligents et de Robotique, Paris, France

**Keywords:** expected utility, hit dispersion, cost-benefit, speed-accuracy, arm reaching

## Abstract

Two basic trade-offs interact while our brain decides how to move our body. First, with the cost-benefit trade-off, the brain trades between the importance of moving faster toward a target that is more rewarding and the increased muscular cost resulting from a faster movement. Second, with the speed-accuracy trade-off, the brain trades between how accurate the movement needs to be and the time it takes to achieve such accuracy. So far, these two trade-offs have been well studied in isolation, despite their obvious interdependence. To overcome this limitation, we propose a new model that is able to simultaneously account for both trade-offs. The model assumes that the central nervous system maximizes the expected utility resulting from the potential reward and the cost over the repetition of many movements, taking into account the probability to miss the target. The resulting model is able to account for both the speed-accuracy and the cost-benefit trade-offs. To validate the proposed hypothesis, we confront the properties of the computational model to data from an experimental study where subjects have to reach for targets by performing arm movements in a horizontal plane. The results qualitatively show that the proposed model successfully accounts for both cost-benefit and speed-accuracy trade-offs.

## 1. Introduction

**Figure 1:**
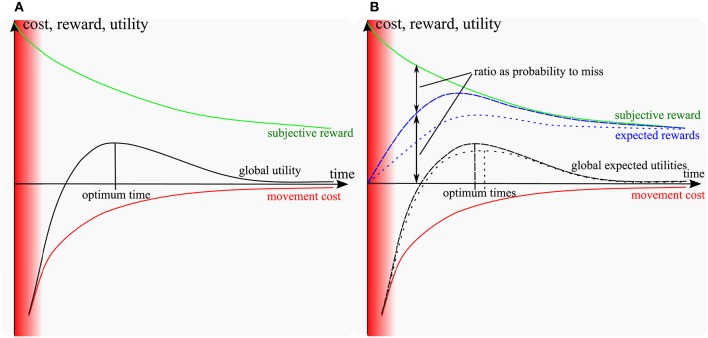
Sketch of models: **(A)** cost-benefit trade-off models (Shadmehr et al., 2010; Rigoux and Guigon, 2012); **(B)** including the probability to miss (proposed model). Red area: unfeasible short times. Green: subjective reward of movement; red: muscular energy cost. Blue and black lines: expected rewards and utilities respectively (dashed for a large target, dotted for a small target). In the standard and proposed models, the subjective utility of getting the reward decreases over time, while slow hitting movements require less muscular effort. As a result, the sum of the reward and the (negative) effort cost reaches a maximum for a certain time (optimum time). The expected reward is the probability-weighted average of the subjective reward over infinitely many repetitions. It converges to the blue line as the number of repetitions increase. The same for the global expected utility. In the proposed model, the probability to hit is null for fast movements and converges to 1 as movements slow down. Furthermore, as movement time increases, the probability to hit increases faster for a large target than for a small one. Therefore, the expected reward matches the subjective reward faster for large targets than for small targets and the optimum time (time at the maximum of global expected utility) is shifted toward longer time for smaller targets, as a result of the speed-accuracy trade-off. Besides, at the optimum time, the probability to miss the target is larger for small targets than for large targets. Note that if the target becomes considerably large compared to the length of the movement, it could affect the cost characteristics. Having a larger target width might decrease the expected effort, because of the biomechanics and anisotropy of inertia of the arm. However, in this study we considered relatively small targets compared to the movement length.

There has been a recent progress in motor control research on understanding how the time of a movement is chosen. In particular, two models proposed an optimization criterion that involves a trade-off between the muscular effort and the subjective value of getting the reward, hence a *cost-benefit trade-off* (CBT) (Shadmehr et al., 2010; Rigoux and Guigon, 2012). On one hand, getting a reward faster requires a larger muscular effort (Young and Bilby, 1993). On the other hand, the subjective value of getting a reward decreases as the time needed to do so is increased (Green and Myerson, 2004). As a result, the net utility consisting of the subjective value minus the muscular effort is optimal for a certain time, as illustrated in Figure 1A. However, these models do not account directly for basic facts about the relation between movement difficulty and movement duration as captured more than 50 years ago by Fitts' law (Fitts, 1954). According to this law, the smaller a target, the slower the reaching movement. This is well explained by the so-called *speed-accuracy trade-off* (SAT) stating that, the faster a movement, the less accurate it is, hence the higher the probability to miss the target. So a subject reaching too fast may not get the subjective value associated to reaching and should slow down. Several studies in the past developed various theories on SAT (Keele, 1968; Schmidt et al., 1979; Crossman and Goodeve, 1983; Meyer et al., 1988; Elliott et al., 2001). Later on, some of the missing aspects were covered by the model of Dean et al. (2007). The key difference to CBT models (Shadmehr et al., 2010; Rigoux and Guigon, 2012) is that, instead of maximizing a reward, this model maximizes a *reward expectation*, i.e., the reward times the probability to get it. However, this model is abstract and it looks for an optimal trade-off between an externally decayed reward and a parametric SAT diagram that relates the probability of missing to movement time. As such, it accounts neither for movement execution, nor for the choice of a motor trajectory and its impact on the cost of movement.

To overcome the limitations of the previously proposed models, we present a new motor control model that is able to account for both CBT and SAT by maximizing the *expected utility* of reaching movements. Unifying both trade-offs allows our model to be the first to account simultaneously for several motor control phenomena related to movement trajectory, velocity profiles and hit dispersion, highlighting the crucial importance of stochastic optimization in the presence of motor noise. Though the model is general, we illustrate its properties in the context of reaching with the arm.

The faster movements tend to be less accurate due to the presence of single-dependant noise in the human motor control system (Schmidt et al., 1979; Meyer et al., 1988; Harris and Wolpert, 1998; Todorov and Jordan, 2002). Our model builds on the stochastic optimal control view of motor control (Meyer et al., 1988; Harris and Wolpert, 1998; Todorov and Jordan, 2002; Todorov, 2004, 2005; Li, 2006), which considers variability as a key ingredient of human movements. In addition, the model adopts an infinite-horizon formulation, like several other models from the literature (Rigoux and Guigon, 2012; Qian et al., 2013).

The general intuition is illustrated in Figure 1B. In the previous CBT models, the target was given as a single point and the movement was considered as always reaching it, irrespective of any target size constraint. In order to fully account for Fitts' law, it is necessary to incorporate the intrinsic dispersion of reaching movements toward a target and the effect of sensory and muscular noise on this dispersion (e.g., Harris and Wolpert, 1998, see Faisal et al., 2008 for a review). As a consequence of noise, movements reaching faster should suffer from a higher dispersion and thus get a lower *probability* of reaching a small target. Thus, instead of optimizing a utility as the sum of a reward and a cost terms as in (1) from Rigoux and Guigon (2012):

(1) $$\begin{array}{l} J\left(\text{u}\right)=\int_{0}^{\infty }e^{-t/\gamma }[\rho R\left({\text{s}}_{t}\right)-\upsilon L\left({\text{u}}_{t}\right)]dt, \end{array}$$

we hypothesize that human motor control optimizes an *expected utility*, that is the above utility times the probability to get it, as captured in (2):

(2) $$\begin{array}{l} J\left(\text{u}\right)={𝔼}_{\text{s},\text{u}}(\int_{0}^{\infty }[e^{-t/\gamma }\rho R\left({\text{s}}_{t}\right)-\upsilon L\left({\text{u}}_{t}\right)]dt), \end{array}$$

where ${𝔼}_{\text{s},\text{u}}\left(\ldots \right)$ denotes the expectation over the random variables ***s*** and ***u***, which is the probability-weighted average of its argument over infinitely many repetitions. In (1) and (2), *t* is time, ${\text{s}}_{t}$ is the current state, ${\text{u}}_{t}$ is the current muscular activation vector, $R\left({\text{s}}_{t}\right)$ is the immediate reward function that equals 1 when reaching the target and is null everywhere else, and $L\left({\text{u}}_{t}\right)$ is the movement cost. As in Rigoux and Guigon (2012) and many other motor control models (e.g., Guigon et al., 2008b), we take $L\left({\text{u}}_{t}\right)=||{\text{u}}_{t}|{|}^{2}$. The meta-parameters of the model, γ, ρ and υ, are explained in section 4.2.1 and given in **Table 3**.

The probability to reach the target can be assumed null for infinitely fast movements with a null duration, and goes to 1 (100%) as movements gets slower. Furthermore, it increases faster for large targets than for small ones. A sketch of the resulting expected reward as a function of movement time is depicted in Figure 1B for a small and a large target. The probability to miss can be inferred in Figure 1B as the ratio between the subjective and the expected rewards, since the expected reward would be equal to the subjective reward if this probability was null.

As can be seen in Figure 1B, if the target is smaller, then the probability to reach it is smaller for a given time, thus the expected reward should itself be smaller. Therefore, the optimum time resulting from the optimal combination of this expected reward with the cost of movement should shift to longer times, which is consistent with Fitts' law.

Furthermore, at the optimum time, the probability to miss the target is larger for small targets than for large targets. This is why subjects miss significantly more often small targets than large targets (see **Figure 4**). Going slower would decrease their probability to miss, but would incur a lower global utility, due to the loss in subjective utility. Explaining this specific phenomenon is a distinguishing property of our model. Besides, other empirical facts resulting from the unification of both trade-offs are studied below.

The proposed computational model was implemented through the simulation of a two degrees-of-freedom (DoFs) planar arm model controlled by 6 muscles, illustrated in **Figure 7**. Critically, the model incorporated delayed feedback and signal-dependent motor noise, accounting for the fact that the motor activation signal descending from the Central Nervous System (CNS) to motoneurons is corrupted with some noise that is proportional to this signal (Selen et al., 2006). An optimization algorithm was used to obtain a controller providing musculars activations to the model. Given stochasticity of the plant and delayed feedback, a state estimation component was required in the control loop (see e.g., Guigon et al., 2008b). We implemented an *ad hoc* state estimator described in section 4.2.3. The goal of the optimized controller was to maximize the cost function given in (4) (see section 4.2.2 for details).

To validate the proposed hypothesis and motor control model, we designed an experimental study where ten subjects had to reach targets displayed on a screen by performing large horizontal arm movements. In order to study the combined effects of the CBT and the SAT on movement trajectory, velocity and hit dispersion, we rewarded the subjects as a function of a number of targets they reached in a limited duration, and we varied the starting point and target size so as to enforce various accuracy constraints in their movements. More precisely, the setup included 15 starting points along three circles at 15, 37.5, and 60% of arm length (see **Figure 5B**) from the target and we used four different target sizes (5, 10, 20, and 40 mm), consistently with the simulation setup. We recorded the reaching hand trajectories of subjects with a dedicated haptic manipulator and their muscular activations through surface electromyography (EMG). To amplify the effect of the cost of motion, the haptic manipulator emulated a viscous media through which the subject had to move the hand. A monitor displayed the current motion from the starting points and a wall where the target was located. From the recorded movement of subjects, we extracted trajectories, movement time, velocity profiles and dispersion of hits on the target. From measured EMG, we obtained muscle activations and calculated the effort related to the arm movement. More details about the experimental study and the computational model are given in section 4.

## 2. Results

We analyzed three main aspects of reaching movements that are most relevant to CBT and SAT. First, we show the velocity profiles. Second, we show the movement reaching times with respect to Fitts' law. Third, we show the hit dispersion diagram that indicates distribution of reaching movement final position on the wall, where the target was located. Each of the three aspects is shown for both simulation and experimental data. Additional supplementary results are presented in Supplementary Data, which show some other details of the reaching movement.

The subjects were given a limited amount of time per session (100 s) in order to be forced to follow CBT. In the available time, on average each subject performed 71.5 ± 13.7 trials and accumulated reward 62.9 ± 13.0 for 5 mm target, 82.9 ± 13.4 trials and reward 81.9 ± 13.4 for 10 mm target, 116.2 ± 17.3 trials and reward 115.9 ± 17.0 for 20 mm target, and 142.6 ± 10.3 trials and reward 140.6 ± 10.3 for 40 mm target.

### 2.1. Velocity profiles

**Figure 2:**
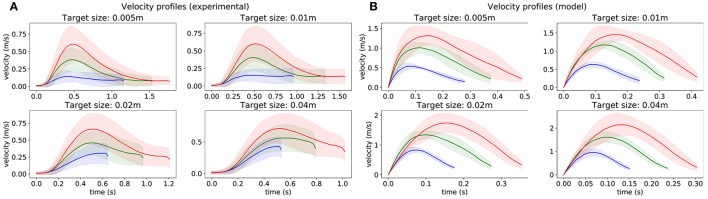
Velocity profiles obtained from 1,500 trajectories starting from 15 points (see Figure 5B), for each target size. **(A)** recorded from subjects. **(B)** obtained from the model. The color of lines depends on the distance between the initial point and the target.

Velocity profiles are depicted in Figure 2. One can see that, for subjects and for the model, movement velocity increases with the distance to the target to compensate for longer distance by permitting higher motor cost in order to reach the target in a reasonable time (i.e., maintain reasonable expected utility according to CBT). This relationship is also evident in Table 1, where the mean peak velocity is increasing with the reaching distance (see columns). Note that the values are normalized to the first value to facilitate an easier comparison. Furthermore, movement time increases when the target is smaller, and also increases with the movement distance, consistently with Fitt's law (i.e., following SAT). This relationship is also evident in Table 2, where the mean movement time is decreasing with the target size (see rows). One can also observe that these relationships are relatively similar between the experimental data and the simulation data.

**Table 1:** Peak velocity relationship to target size and distance (normalized to the first value).

		**Target [m] - experimental**				**Target [m] - model**			
		0.005	0.01	0.02	0.04	0.005	0.01	0.02	0.04
Distance	Short	1.00	1.08	2.13	2.98	1.00	1.20	1.56	1.74
	Med.	2.69	2.83	3.20	3.90	2.07	2.18	2.43	2.90
	Long	4.20	4.14	4.58	4.93	2.45	2.68	3.36	3.93

**Table 2:** Movement time relationship to target size and distance (normalized to the first value).

		**Target [m] - experimental**				**Target [m] - model**			
		0.005	0.01	0.02	0.04	0.005	0.01	0.02	0.04
Distance	Short	1.00	0.83	0.56	0.46	1.00	0.82	0.62	0.54
	Med.	1.32	1.15	0.83	0.69	1.24	1.12	0.97	0.86
	Long	1.53	1.35	1.04	0.88	1.66	1.49	1.22	1.09

In addition, these profiles resemble asymmetric bell-shaped trajectories with the peak velocity lying early in the movement, which corresponds to the established studies from the literature (Plamondon, 1991). The time of this peak occurs earlier when the target is smaller, and later for longer movements. Additionally, the endpoint velocity does not differ much depending on the length of the movement, but is higher for larger targets.

There are also some discrepancies between subjects and the model. The most obvious is that the model is much faster than the subjects who also slow down the motion sooner than the model, resulting in a more pronounced peak. These discrepancies are further discussed in section 3.4.1.

### 2.2. Fitts' law

Fitts' law states that the movement time (MT) is linear in its index of difficulty (ID), this index being larger for longer movements and smaller targets. The equation that describes the Fitts' law is

(3) $$\begin{array}{l} \text{MT}=a.\underset{\text{ID}}{\underset{︸}{\log_{2}\left(\frac{D}{W}\right)}}+b, \end{array}$$

where *D* is the length of the movement (denoted with *A* for amplitude in other papers), *W* is the width of the target and *a* and *b* are the linear coefficients. This law was initially studied for one dimensional movements, and then extended for many other contexts (Soechting, 1984; Bootsma et al., 1994; Laurent, 1994; Plamondon and Alimi, 1997; Smyrnis et al., 2000; Bootsma et al., 2004).

**Figure 3:**
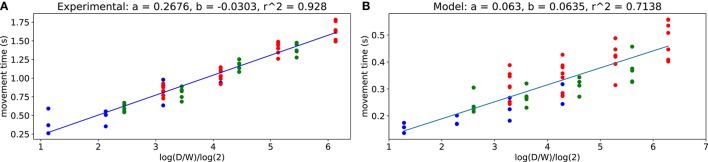
Fitts' law. **(A)** obtained from all subjects trajectories. **(B)** obtained from the model. Each dot corresponds to an average for each target size and target distance pair, obtained either over all subjects or over 1,500 model trajectories.

From the trajectory data, we computed *ID* values for different distances *D* and target widths *W* using (3). Figure 3 shows the resulting movement time *MT* over *ID* for the subject and for the model. One can see that the experimental data are strongly consistent with Fitts' law (*r*^2^ > 0.9) which also holds for the computational model (*r*^2^ > 0.7).

The obtained values of *b* are quite similar between the model and the experimental data, corresponding to a close-to-null offset. However, the values of *a* do not match. First, as outlined in section 2.1, the movements from the computational model are faster than those of subjects (see section 3.4.1 for discussion). Besides, according to the literature, the values of *a* vary widely across subjects (Crossman and Goodeve, 1983; Scott MacKenzie, 1989).

### 2.3. Hit dispersion

**Figure 4:**
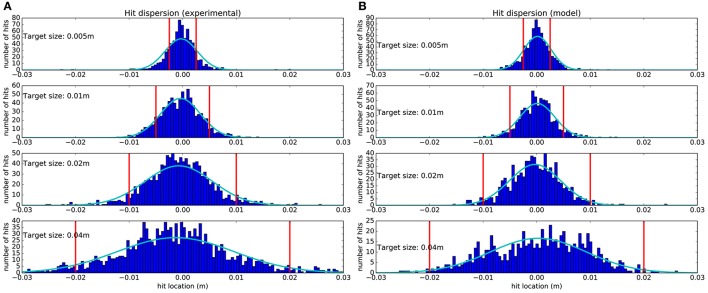
Hit dispersion diagram obtained from 1,500 trajectories starting from 15 points (see Figure 5B), for each target size. **(A)** Recorded from subjects. **(B)** Obtained from the model. The vertical red lines denote the lateral target boundaries. Dark blue histograms are obtained by counting all the trajectories that hit the target within a 0.5 millimeter range. Light blue Gaussians are fitted to the histograms.

The hit dispersion resulting from our study is shown in Figure 4. One can observe a good match between experimental hit dispersion and the one obtained from the model. The Kullback–Leibler divergence was: 0.032, 0.029, 0.069, and 0.043, respectively for each target. This good match is a distinguishing feature of our model as, to our knowledge, it is the first that can reproduce this property of human reaching movements. Nevertheless, the positive correlation between hit dispersion and target width is coherent with the minimal intervention principle (Todorov and Jordan, 2002).

One can observe that subjects and the model tend to hit more in the center of target, which is in first approximation a good way to maximize the expected utility. The mean location of the fitted Gaussians' peaks with respect to the target center and normalized to the target size for experimental data is at −5.4 ± 2.5% and for model is at 0.63 ± 1.5%. Furthermore, when the target is smaller, dispersion is reduced to increase the probability of reaching the target successfully. This reduced dispersion is obtained at the price of a longer movement time, as illustrated in Figure 3, as a result of SAT.

However, the probability to miss the target is never null, neither for the subjects nor for the model. Since the experimental task included a limitation on the available time to perform the task, it was more optimal to pay the price of a few failed movements than to move slow enough to succeed every time. This resulted in CBT, as moving slowly would mean spending too much of the limited time and consequently missing an opportunity to get a larger cumulative reward.

## 3. Discussion

As outlined in the introduction, the model presented in this paper assumes that the CNS optimizes the *expected utility* of reaching movements. As Equation (2) shows, this expected utility is a function of three factors: the discounted reward resulting from reaching, which is itself a decreasing function of movement time, the probability to get this reward, which decreases with hit dispersion, and the cost of movement, which depends on the movement trajectory and timing. In order to optimize the expected utility, the CNS must find an optimal trade-off between these competing factors, by adjusting the instantaneous muscular activations.

We qualitatively analyzed three main aspects of reaching movements that are most relevant to CBT and SAT. First, the results show that the proposed model accounts for CBT as the velocity of movement is higher for more distant targets. This suggests that higher cost is permitted by the model/CNS to compensate for longer distances in order to reach the target in a reasonable time. Second, the results show that our model accounts for SAT by following Fitts' law, as the reaching time increases while the target size decreases to compensate for the required higher accuracy. Third, the results show that the model accounts for stochasticity of movement due to motor noise, as the target is sometimes missed and the hit position frequency on the target follows Gaussian distribution. Thus, even if our model does not quantitatively account for the reaching time of subject (see section 3.4.1 for discussion), the good qualitative match between experimental results and the behavior of our model strongly suggests that subjects do optimize their reaching time with respect to the global expected utility of their movement, i.e., taking the probability to miss into account. Additional results are in Supplementary Data and which point out that the proposed model can account for several other phenomena observed in the experimental data, such as: asymmetric muscle effort cost with respect to the initial point, and tendency to hit the target perpendicularly.

### 3.1. Our model simultaneously accounts for both CBT and SAT

The models presented in Shadmehr et al. (2010) and Rigoux and Guigon (2012) only related discounted reward and movement cost to explain the time of movement. In particular, motor control results in Rigoux and Guigon (2012) were obtained in the absence of sensory and motor noise. This model could explain several well-established motor control facts, as expected from the close relationship to previous optimal control models (Gordon et al., 1994; Shadmehr and Mussa-Ivaldi, 1994; Todorov and Jordan, 2002; Guigon et al., 2007, 2008a; Liu and Todorov, 2007), as well as several phenomena specific to CBT tasks (Watanabe et al., 2003; Rudebeck et al., 2006). However, the model in Rigoux and Guigon (2012) could not provide a direct account of phenomena relying on the stochasticity of the motor system, such as Fitts' law. Actually, their model provided an indirect account of Fitts' law (see, Rigoux and Guigon, 2012). For obtaining these results, the authors had to estimate dispersion as a function of velocity considering a constant velocity over the movement, and they reconstructed the relationship between the index of difficulty and the movement time based on a single starting point and the size of a unique target that would match this estimated dispersion (personal communication).

On the other hand, the model of Dean et al. only related discounted reward and accuracy, without consideration for movement cost (Dean et al., 2007). An abstract SAT model was directly fitted to human movement data, taking Fitts' law as a prior rather than explaining it. As such, the model could not account for several motor control phenomena related to the cost of movement.

In section 2.2, we have shown that our model accounts for Fitts' law. In contrast with the model of Dean et al. (2007), in our model the hit dispersion is measured as an effect of motor noise and imperfect state estimation along optimized trajectories, rather than inferred based on a given SAT model. Thus, one of the contributions is a model that addresses the more global inter-relationship between the discounted reward, the probability to reach, and the movement cost through an optimality criterion that accounts for the motor strategy of human subjects in this multi-dimensional choice space. Furthermore, simultaneously taking into account the three factors above endows our model with further properties, resulting in the contributions highlighted below.

### 3.2. Our model accounts for hit dispersion

In Figure 4, we observe that hit dispersion is smaller for smaller targets, even if there are still some failed movements. Our model puts forward two explanations on how the CNS might do so. First, the hit velocity is lower for smaller targets. Up to a certain level, motor noise can be reduced without slowing down by generating less co-contraction. However, below a certain threshold, less muscular activation implies a slower movement. Thus, the CNS achieves higher accuracy just by arriving slower at the target. Second, we observe in Figure 2 that subjects start slowing down earlier when the target is smaller. By doing so, they give more time to the state estimation process to accurately estimate the end effector position, which is another way to reduce hit dispersion. It is quite likely that both mechanisms contribute to the necessary reduction in hit dispersion.

Recently, a study by Wang et al. (2016) empirically observed that human subjects preferred to execute the reaching movement with a time higher than the time at which the best endpoint variability was achieved. Based on this, they hypothesized that the human CNS tends to minimize both effort and endpoint variability. While their study excluded the visual feedback, their results are generally in line with the results of our study. However, Wang et al. (2016) did not devise any mathematical model to replicate their hypothesis.

### 3.3. Our model accounts for asymmetric bell-shaped velocity profiles

The hit dispersion observed in Figure 4 results from motor noise and imperfect state estimation. As a consequence of motor noise being proportional to muscular activation, one way to decrease motor noise is to decrease muscular activation. In the case of the minimum intervention principle (Todorov and Jordan, 2002), it results in the fact that the brain will not correct an error which is not related to task achievement. In the case of reaching movements, it will also result in a tendency to move fast earlier to be accurate later, when accuracy matters. Additionally, under the assumption that more accurate state estimation takes more time, a slower movement provides a better opportunity for state estimation to compensate for delayed feedback about the current position of the end effector. Taken together, both phenomena drive controller optimization toward generating less velocity by the end of the movement for a smaller target. So, one way to make sure to hit a small target would be to perform a slow reaching movement.

However, a slower movement results in a more discounted reward, thus the movement should nevertheless be as fast as possible. As a consequence, the best way to optimize reaching accuracy under temporal constraints is to be faster in the beginning of the movement and slower in the end. Such asymmetry was also observed in the literature (MacKenzie et al., 1987; Jean and Berret, 2017). This phenomenon can be attributed to the use of visual feedback, as it has been shown that the peak in velocity profiles occurs earlier in the movement when the visual feedback is available compared to when no visual feedback is available (Hansen et al., 2006; Burkitt et al., 2013). Thus, velocity profiles should be bell-shaped and asymmetric, as visible in Figure 2. By contrast, the model of Dean et al. assumes constant velocity (Dean et al., 2007) and the one from Rigoux and Guigon are bell-shaped, but not asymmetric (Rigoux and Guigon, 2012).

### 3.4. Potential limitations

#### 3.4.1. Discrepancy in movement times between experimental and simulation data

The movement time discrepancy may be due to various factors:

1. The presence of the haptic manipulator and an additional external damping, which significantly slows down the motion of the subjects and which is not accounted for by the model,
2. Inaccuracies in the arm model, which does not account for the effects of muscular friction that slows down the arm,
3. Inaccuracies in the state estimation process. As for the latter, if estimation is too good in the model, the simulated arm may go faster without generating too much inaccuracy.

However, the purpose of this study was not to precisely predict the movement times of the subjects, but to show that the model can account for both CBT and SAT. For clarity and generality, the model was simplified and did not include the external damping of the haptic manipulator. Therefore, the movement times of the model do not correspond to the movement times of the subjects, as the model could move faster due to the less resistance. In particular, any significant addition external damping increases the movement time due the increased effort (Tanaka et al., 2006), therefore such time movement discrepancy is in accordance with the literature.

Including more detailed or better matching models at various stages/aspects could indeed make the data look closer to each other. However, the primary focus of this study was to validate the hypothesis that the model is able to account for both SAT and CBT. In addition, a general model should be able to account for SAT and CBT independently of conditions. Therefore, trying to precisely match the simulations to any particular experimental condition (e.g., external damping) would not help to show such generality, nor would provide any additional validation of unification hypothesis.

#### 3.4.2. Various discounting of reward through time

The existing study did not explore all design choices of how the model should discount the reward through time that has been studied in the literature. The debate between diverse discounting approaches is a long standing one (see e.g., Green and Myerson, 1996; Berret and Jean, 2016). The model of Rigoux and Guigon (2012) uses an exponential discounting of the reward through time. Alternative models suggest linear (Hoff, 1994), quadratic (Shadmehr et al., 2010), or hyperbolic (Shadmehr et al., 2010) discounting approaches. The latter is also in line with studies of many other authors (e.g., Prévost et al., 2010). More recently, using an inverse optimal control approach, it was determined that the experimental “cost of time” for reaching movements would rather follow a sigmoidal function (Berret and Jean, 2016).

#### 3.4.3. Two-component reaching strategy

Several past studies observed that human reaching movements tend to follow two distinct phases (Woodworth, 1899; Meyer et al., 1988; Elliott et al., 2001, 2010). The initial phase, which usually constitutes most of the movement, is rapid and relatively predictable. In the second phase, when the target is approached, the movement is slowed down and the time-displacement profiles often have discontinuities, which reflect modifications to the trajectory (Elliott et al., 2001). While we observed a similar behavior in our subjects, the presented model is not able to explicitly account for such two-component reaching strategy.

#### 3.4.4. Flat target

In the classic Fitts's law, the target surface is assumed to be oriented perpendicularly with respect to the movement. In our study the target was flat and the hits could be done from different angles. Some insights about this aspect are presented in Supplementary Material. The experimental data showed that subjects hit the target from different angles, but the majority of hits were closer to the perpendicular direction. To account for this observed aspect, we extended the proposed model to include the perpendicularity cost [see (4) in section 4.2.1 for details].

#### 3.4.5. Reliance on selected parameters and optimization process

The resolution of the optimization problem to acquire the controller relies on extensive numerical experiments with several tunable parameters. The optimization process can be time-consuming and selecting the suitable parameters is essential in acquiring a good solution. This can be viewed as one of the disadvantages of the proposed method.

### 3.5. Future work

Beyond better fitting the data and studying the model properties through systematic variations of the meta-parameters, the main line in our research agenda consists in shifting from a motor control perspective to a motor learning perspective by focusing on the optimization process itself. Under a motor learning perspective, we might for instance study the evolution of trajectories, co-contraction, velocity profiles, etc. along the training process. We might also check whether our model accounts for already published motor learning studies such as the work of Izawa et al. (2008) or Diedrichsen et al. (2010). We might explore different discounting of the reward through time from the literature (Berret and Jean, 2016). In addition, we will try to extend the existing model to incorporate multiple-component reaching strategy (Elliott et al., 2017).

## 4. Methods

### 4.1. Experimental methods

#### 4.1.1. Participants

Ten healthy male volunteers participated in the study. Their average age was 23.0 years (SD = 2.7 years), height 178.8 cm (SD = 4.1 cm) and body mass 77.4 kg (SD = 5.8 kg). All subjects were right-handed. Exclusion criteria were neurological, vestibular, locomotion, visual disorders and recent limb injuries (self-reported). This study was carried out in accordance with the recommendations of National Medical Ethics Committee Slovenia (NO. 112/06/13) with written informed consent from all subjects. All subjects gave written informed consent in accordance with the Declaration of Helsinki. The protocol was approved by the National Medical Ethics Committee Slovenia (NO. 112/06/13).

#### 4.1.2. Apparatus

In most experiments where a subject has to reach a target, either in monkeys (e.g., Kitazawa et al., 1998) or humans (Trommershäuser et al., 2003, 2009; Battaglia and Schrater, 2007; Dean et al., 2007; Hudson et al., 2008), the target is displayed on a vertical screen and the subject performs a trajectory in the coronal or horizontal plane that the screen intercepts. Our experimental apparatus reproduces such a scene in the horizontal plane.

**Figure 5:**
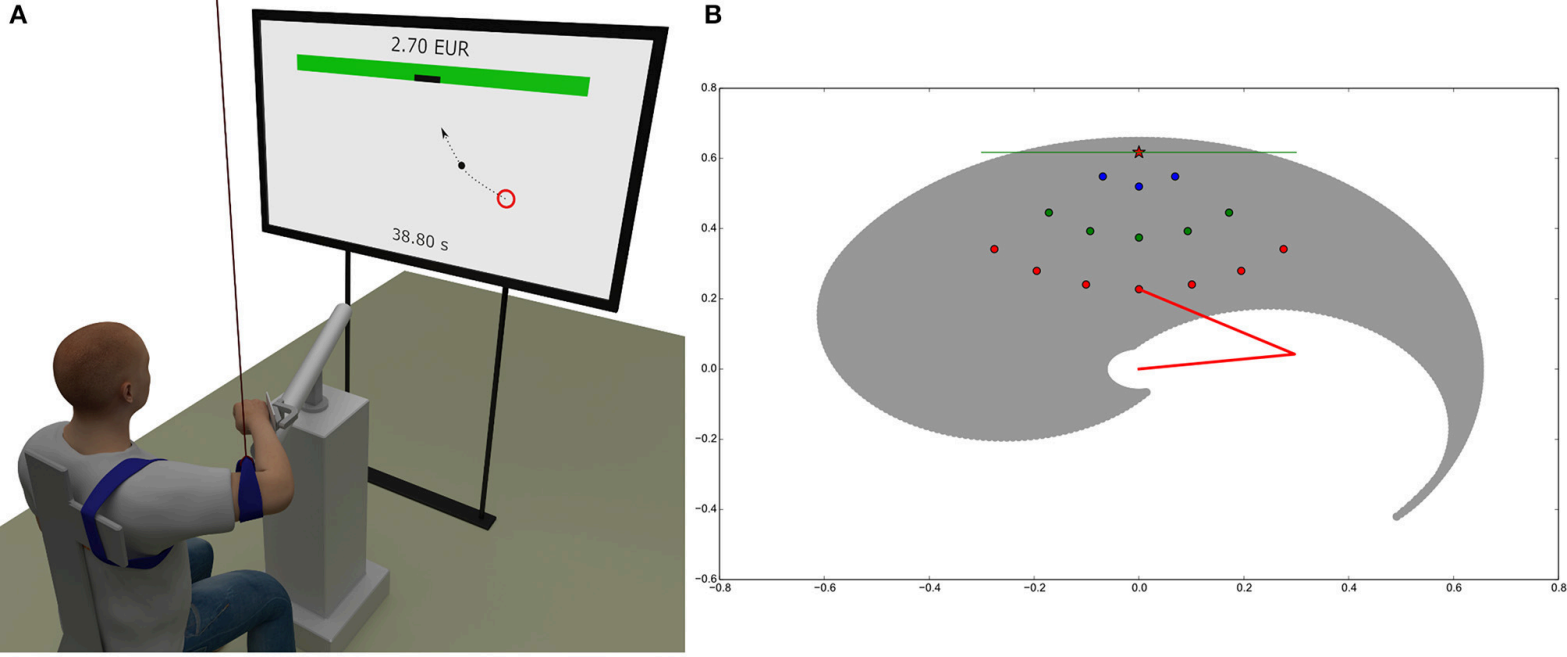
Experimental and simulation setup. **(A)** Subjects sat on a chair in front of a TV screen with the right wrist connected to the haptic manipulator that constrained the motion of the right hand to the horizontal plane at the shoulder height. The screen displayed the current position of the subject's hand, the randomly occurring initial circular areas, the target on the virtual wall and gave feedback about the remaining time and the cumulative reward. **(B)** Simulated arm workspace and distribution of the 15 initial circular areas relative to the target on the wall. The reachable space is delimited by a spiral-shaped envelope (gray). The two segments of the arm are represented by red lines. The screen is represented as a green line positioned at *y* = 0.6175*m* and the target center as a red star. The origin of the arm is at *x* = 0.0, *y* = 0.0. Initial areas are organized into three sets at different distances to the target, corresponding to 15% (3 blue circles), 37.5% (5 green circles), and 60% (7 red circles). The color code of the dots depending on the distance to the target is reused throughout the paper (in Fitts' law and velocity profiles diagrams).

As depicted in Figure 5A, subjects sat on a chair in front of a TV screen and a 3 axes haptic manipulator (HM) (Haptic master Mk2, MOOG, Nieuw-Vennep, The Netherlands). The TV screen was located 2 m in front of the chair backrest. The subject's right wrist was immobilized and connected to the HM by means of the gimbal mechanism (ADL gimbal mechanism, MOOG, Nieuw-Vennep, The Netherlands). HM constrained the motion of the subject's right hand (hand from here on) to the horizontal plane at the height of the subject's shoulders. The subject's right elbow was suspended from the ceiling by a long string in order to compensate for the effect of gravity and to restrain the motion of the arm to the horizontal plane. In order to fix the position of the shoulders, the trunk of the subject was immobilized by tying it to the backrest of the chair.

Details about the performed trajectories are shown in Figure 5B. Subject's were allowed to move the hand from 15 initial circular areas with 10 mm diameter spread in front of them to a single target that was located symmetrically to their right shoulder on a virtual wall at a distance equal to 95% of their arm length (defined as the distance between shoulder and wrist). The initial areas were spread on three arcs with their center on the target on the virtual wall. The radius of the first arc was equal to 15% of the arm length and included 3 initial areas, the radius of the second arc was equal to 37.5% of the arm length and included 5 initial areas, and the radius of the third arc was equal to 60% of the arm length and included 7 initial areas. The initial areas on each arc were placed symmetrically with respect to the target on the virtual wall and were spread within the ±45°. The initial areas were spread in the region well within the area of arm motion where the passive joint torques are negligible.

The size of the target on the virtual wall was either 5, 10, 20, or 40 mm wide. The position of the current initial area, the position of the target and the position of the subject's hand were drawn in 2D perspective on the screen in real time.

The experiment was divided into five parts corresponding to the four randomly selected sizes of the target, preceded by a familiarization trial with the target size of 20 mm. In each part, the randomly selected initial position from where the subject had to perform the motion was indicated in red. When the subject reached and stood within the initial position for more than 1 s, the color of the patch changed from red to green. This allowed the execution of the complete behavioral neuro-cognitive processes responsible for the SAT (Perri et al., 2014). When the patch turned green, the subject was indicated that the motion could be started. The time of the motion from the moment when the hand left the initial circular patch to the moment when the hand touched the virtual wall was recorded and subtracted from the total time of 100 s. The remaining time was displayed at the bottom of the screen. Success in hitting the target on the virtual wall was clearly indicated by changing the color of the virtual wall from gray to green for 1*s*. Besides, the subject obtained a money award of 2.5 euro cents. If the target was missed, the virtual wall turned red and no reward was given to the subject. The cumulative reward was displayed on top of the screen using large bold fonts.

The task of the subject was to obtain as high reward as possible in the given time. The cost of movement for each starting point was calculated by using muscle activity measurements obtained from EMG. The results regarding the movement cost are presented in Supplementary Material. To amplify the effect of the cost of motion, the haptic manipulator emulated a viscous media through which the subject had to move the hand. The coefficient of viscous friction was set to 30*Nm*^−1^*s*. In the analysis, the muscular activations necessary to compensate for the friction of the haptic manipulator at the end-effector were removed by estimating them through the arm model described in section 4.2.5 and a model of the haptic manipulator friction.

### 4.2. Computational methods

The computational model consists of a simulation set-up, an arm model, a state estimator, a set of controllers and a way to optimize these controllers. All the meta-parameters of these various components are summarized in Table 3, apart from arm model parameters which are given in section 4.2.5. Though the model is formally described using continuous time, in all the computational methods time is discretized with a time step δ_*t*_ = 0.002*s*.

**Table 3:** Meta-parameters of the computational model.

	ρ	Immediate reward factor	3,000
Cost	υ	Effort term factor	1
function	γ	Discount factor	0.6
	σ	Initial covariance factor	0.01
CMA-ES	*max*_*iter*	Maximum number of iterations	5,000
	*popsize*	Population size	30
	*repet*	Number of repetitions	50
	κ	Multiplicative muscular noise	0.3
State	Δ	Sensory delay in time steps	10
estimation	*k*_1_	Open-loop term factor in state estimation	0.2
	*k*_2_	Closed-loop term factor in state estimation	1.0
Controller	*N*_*h*_	Number of neurons in hidden layer	10

#### 4.2.1. Specific computational model

The model described in the introduction was general. Here, we give a more specific account of the computational model of arm reaching movement which is actually used in the simulations.

Instead of (2), the complete cost function that we optimize is

(4) $$\begin{array}{l} J\left(\text{u}\right)={𝔼}_{\text{s},\text{u}}(\int_{0}^{\infty }[e^{-t/\gamma }\rho R\left({\text{s}}_{t}\right)-\upsilon L\left({\text{u}}_{t}\right)]dt+C\left({\text{s}}_{f}\right)), \end{array}$$

where the perpendicularity cost $C\left({\text{s}}_{f}\right)$ accounts for the tendency of subjects to hit the target perpendicularly and penalizes the scalar product between a vector colinear to the target and the Cartesian velocity of the end effector when the arm hits the screen. The necessity of this additional term is discussed in Supplementary Material.

There are three meta-parameters in (4): the continuous-time discount factor γ accounts for the “greediness” of the controller, i.e., the smaller γ, the more the agent is focused on short term rewards, ρ is the weight of the reward term and υ is the weight of the effort term. Their value is given in Table 3.

#### 4.2.2. Controllers and optimization

In order to evaluate the criterion defined in (4), we need a controller which optimizes it. Actually, the optimal control problem arising from a cost function including an expectation cannot be solved analytically. The utility expectation itself as defined in (4) must be estimated empirically as an average of the return over a set of attempts to hit the target (these attempts are called “rollouts” hereafter). The more rollouts, the better the estimate but we have to limit their number because computation takes time.

We thus rely on a stochastic optimization process, where a controller is represented as a parametric function whose parameters are tuned to optimize (4). The class of functions considered here consists of multi-layer neural networks with one input layer, one output layer and a hidden layer. In order to facilitate optimization by decoupling the parameter optimization problems for each trajectory, we define one such network for each starting point.

These controllers take the state of the system as input and provide muscular activations for all muscles in the arm model. As described in section 4.2.4, states are 4D and muscular activations are 6D, thus we have 4 + *N*_*h*_ + 6 neurons in the networks, where the meta-parameter *N*_*h*_ is the number of neurons in the hidden layer.

All networks are initialized randomly with all weights and biases taken in [0, 0.1]. They are then optimized with respect to the approximated utility expectation described above using a state-of-the-art black-box optimization tool named CMA-ES (Hansen et al., 2003). Given an initial random controller, CMA-ES optimizes its parameters with local stochastic search. New rolloutsare performed with varying parameters for all parameters around those of the current controllers, and the parameters that give rise to a better performance with respect to the cost function (4) are retained in the new current controller. In practice, the parameters are the weights and biases of each neuron in the networks.

CMA-ES comes with four meta-parameters: the initial size σ of a covariance matrix used for exploration, the size of the population *popsize*, the number of repetitions *repet* for each trajectory to get a decent estimation of the reward expectation and the maximum number of iterations *max*_*iter*. The values of these meta-parameters are given in Table 3.

#### 4.2.3. State estimation

**Figure 6:**
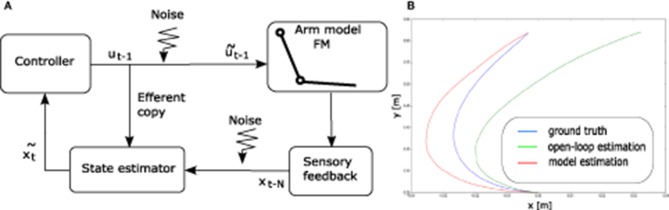
**(A)** A standard schematic view of human motor control (see e.g., Scott, 2004). **(B)** Illustration of the state estimation dynamics. The open-loop term (in green) diverges from the perturbation-free “ground truth" trajectory (in blue). The model state estimation (in red) diverges in the accelerating phase of the movement, but the closed-loop term makes it converge again toward ground truth when the end-effector decelerates.

A widely accepted overview of the human motor control loop is depicted in Figure 6A. According to this view, human movements are performed in closed loop. However, the sensory feedback being delayed, the control loop has to rely on state estimation to be stable (see Figure 6B).

Our implementation of the state estimator contains an estimate of the current state $\tilde{x_{t}}$ of the arm. The initial estimated state $\tilde{x_{0}}$ is the initial state of the arm *x*_0_. Then, at each time step *t*, this estimated state is updated by the combination of two terms ${\tilde{x}}_{t}^{1}$ and ${\tilde{x}}_{t}^{2}$.

The first term corresponds to an open-loop estimation. At each time step, it simply updates the estimated state by applying the forward model of the arm to the previous estimated state, given the efferent copy of the motor command sent to the arm (before adding motor noise), i.e.,

$${\tilde{x}}_{t}^{1}=FM\left({\tilde{x}}_{t-1}^{1},u_{t-1}\right).$$

This term being open-loop and the motor command being different from the noisy command that has actually been applied to the arm, it generates an estimated state trajectory that may eventually drift away from the actual trajectory of the end effector.

The second term is in charge of closing the estimation loop by making profit of the delayed sensory feedback. The actual state of the arm *x*_*t*−Δ_ is available after a sensory delay of Δ time steps.

A first, naive approach to this second term is the following. Given this known state *x*_*t*−Δ_ and the Δ efferent copy of the commands *u*_*t*−Δ_ to *u*_*t*−1_ sent to the arm in between, one can infer an estimate of the current state ${\tilde{x}}_{t}^{2}$ by simply applying the forward model of the arm Δ times starting from *x*_*t*−Δ_ given the sequence of commands, using

$$\begin{array}{l} {\tilde{x}}_{t-\Delta -1}^{2}=FM\left(x_{t-\Delta },u_{t-\Delta }\right),\\ {\tilde{x}}_{t-\Delta -2}^{2}=FM\left({\tilde{x}}_{t-\Delta -1}^{2},u_{t-\Delta -1}\right),\\ \ldots \\ {\tilde{x}}_{t}^{2}=FM\left({\tilde{x}}_{t-1}^{2},u_{t-1}\right). \end{array}$$

Again, the obtained state estimation ${\tilde{x}}_{t}^{2}$ is not perfectly accurate given that the available efferent copy of the commands do not incorporate the applied motor noise. However, this term counterbalances the drifting tendency of the open-loop term because it updates the current estimate from some delayed ground truth. This approach neglects sensory noise. As a result, it is simpler than the standard state estimation model using an extended Kalman filter, as described in Guigon et al. (2008b).

However, this simpler approach still suffers from its computational cost, because it requires Δ iterations of the forward model of the arm. In order to improve the computational efficiency of the model, we call upon a neural network to replace these Δ iterations by a single function call. The neural network learns to predict the current estimated step given the delayed ground truth *x*_*t*−Δ_ and the Δ efferent copies that were received in between.

We made sure through dedicated simulations (not shown) that the learned neural network provides a reasonably accurate estimate of the current state, corresponding to what Δ iterations of the forward model would have inferred, at a much lower computational cost and with an increased biological implementation plausibility.

Finally, we combine the open-loop and the closed loop term by a weighted summation, using

$$\tilde{x_{t}}=\left(k_{1}{\tilde{x_{t}}}^{1}+k_{2}{\tilde{x_{t}}}^{2}\right)/\left(k_{1}+k_{2}\right).$$

The delay in number of time steps Δ as well as coefficients *k*_1_ and *k*_2_ are meta-parameters described in Table 3. They are tuned so that the estimated state can significantly differ from the true state when the arm is moving fast, but can become more accurate again when the arm slows down, so as to give a chance to hit the target. This tuning process was performed empirically over a small set of trajectories before starting to optimize the controller parameters.

#### 4.2.4. Simulation set-up

The state-space consists of the current articular position **q** of the arm and its current articular speed $\dot{q}$. The state $s=\left(q,\dot{q}\right)$ has a total of 4 dimensions. The initial state is defined by null speed and a variable initial position. The positions are bounded to represent the reachable space of a standard human arm, with *q*_1_ ∈ [2.6, −0.6] and *q*_2_ ∈ [3.0, −0.2], as shown in Figure 5B. The action-space consists of an activation signal for each muscle, resulting in a total of 6 dimensions.

The target is defined as an interval of varying length around (*x* = 0, *y* = 0.6175*m*). The movement is stopped once the line *y* = 0.6175*m* has been crossed, and the intersect between the trajectory and this line is computed to determine whether the target was hit. The reward for immediately hitting the target without taking incurred costs into account depends on the meta-parameter ρ (see Table 3).

#### 4.2.5. Arm model

**Figure 7:**
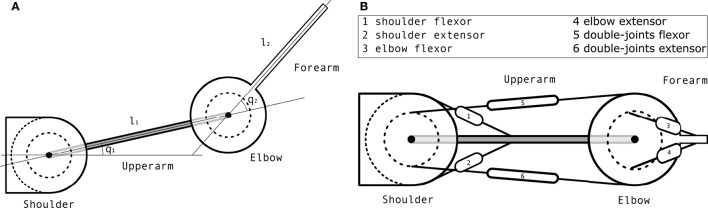
Arm model. **(A)** Schematic view of the arm mechanics. **(B)** Schematic view of the muscular actuation of the arm, where each number represents a muscle whose name is in the box.

The plant is a two degrees-of-freedom (DoFs) planar arm controlled by 6 muscles, illustrated in Figure 7. There are several such models in the literature. The model described in Kambara et al. (2009) lies in the coronal plane so it takes the gravity force into account. Most other models are defined in the horizontal plane and ignore gravity effects. They all combine a simple two DoFs planar rigid-body dynamics model with a muscular actuation model. The differences between models mostly lie in the latter component. Our muscular actuation model is taken from Katayama and Kawato (1993) (pp. 356–357) through (Mitrovic et al., 2008). It is a simplified version of the one described in Li (2006) in the sense that it uses a constant moment arm matrix **A** whereas Li (2006) is computing this matrix as a function of the state of the arm. This arm model is used in simulation through a standard Euler integration method.

The rigid-body dynamics equation of a mechanical system is

(5) $$\begin{array}{l} \ddot{q}=M{\left(q\right)}^{-1}\left(\tau -C\left(q,\dot{q}\right)-g\left(q\right)-B(\dot{q})\right) \end{array}$$

where **q** is the current articular position, $\dot{\text{q}}$ the current articular speed, $\ddot{\text{q}}$ the current articular acceleration, **M** the inertia matrix, **C** the Coriolis force vector, **τ** the segments torque, **g** the gravity force vector and **B** a damping term that contains all unmodelled effects. Here, **g** is ignored since the arm is working in the horizontal plane.

The inertia matrix is computed as $M=\left[\begin{array}{cc} k_{1}+2k_{2}\cos(q_{2}) & k_{3}+k_{2}\cos(q_{2})\\ k_{3}+k_{2}\cos(q_{2}) & k_{3} \end{array}\right],$ with $k_{1}=I_{1}+I_{2}+m_{2}l_{1}^{2}$, *k*_2_ = *m*_2_*l*_1_*s*_2_ and *k*_3_ = *I*_2_, where *m*_*i*_ is the mass of segment *i*, *l*_*i*_ the length of segment *i*, *I*_*i*_ the inertia of segment *i* and *s*_*i*_ the distance from the center of segment *i* to its center of mass. The value of all these parameters is given in Table 4. They have been taken from the literature.

**Table 4:** Arm parameters.

*m*_1_	Arm mass (*kg*)	1.4
*m*_2_	Forearm mass (*kg*)	1.1
*l*_1_	Arm length (*m*)	0.3
*l*_2_	Forearm length (*m*)	0.35
*I*_1_	Arm inertia (*kg*.*m*^2^)	0.025
*I*_2_	Forearm inertia (*kg*.*m*^2^)	0.045
*s*_1_	Distance from the center of segment 1 to its center of mass (*m*)	0.11
*s*_2_	Distance from the center of segment 2 to its center of mass (*m*)	0.16

The Coriolis force vector is $\text{C}=\left[\begin{array}{c} -\dot{q_{2}}\left(2\dot{q_{1}}+\dot{q_{2}}\right)\\ {\dot{q_{1}}}^{2} \end{array}\right]k_{2}\sin\left(q_{2}\right).$

The damping matrix **B** is defined as $B=\left[\begin{array}{cc} b_{1} & b_{2}\\ b_{3} & b_{4} \end{array}\right]\dot{q}=\left[\begin{array}{cc} 0.05 & 0.025\\ 0.025 & 0.05 \end{array}\right]\dot{q}.$

The computation of the torque **τ** exerted on the system given an input muscular actuation **u** is as follows. First, the muscular activation is augmented with Gaussian noise using $\tilde{u}=u_{t}\times \left(1+\kappa N(0,I)\right)$, where × refers to the element-wise multiplication and **I** is a 6 × 6 identity matrix. Then, the input torque is computed as $\tau ={\text{A}}^{⊤}\left({\text{f}}_{\text{max}}ũ\right)$, where the moment arm matrix **A** is defined as

$$\begin{array}{l} {\text{A}}^{⊤}=\left[\begin{array}{cccccc} a_{1} & a_{2} & a_{3} & a_{4} & a_{5} & a_{6}\\ a_{7} & a_{8} & a_{9} & a_{10} & a_{11} & a_{12} \end{array}\right]\\ =\left[\begin{array}{cccccc} 0.04 & -0.04 & 0.0 & 0.0 & 0.028 & -0.035\\ 0.0 & 0.0 & 0.025 & -0.025 & 0.028 & -0.035 \end{array}\right], \end{array}$$

and the matrix of the maximum force exerted by each muscle is defined as

$$\begin{array}{l} {\text{f}}_{\text{max}}=\left(\begin{array}{cccccc} 700 & 0 & 0 & 0 & 0 & 0\\ 0 & 382 & 0 & 0 & 0 & 0\\ 0 & 0 & 572 & 0 & 0 & 0\\ 0 & 0 & 0 & 445 & 0 & 0\\ 0 & 0 & 0 & 0 & 159 & 0\\ 0 & 0 & 0 & 0 & 0 & 318 \end{array}\right). \end{array}$$

The nomenclature of all the parameters and variables of the arm model is given in Table 5.

**Table 5:** Nomenclature of the arm model parameters.

*m*_*i*_	Mass of segment *i* (*kg*)
*l*_*i*_	Length of segment *i* (*m*)
*I*_*i*_	Inertia of segment *i* (*kg*.*m*^2^)
*s*_*i*_	Distance from the center of
	Segment *i* to its center of mass (*m*)
***A***	Moment arm matrix (∈ ℝ^6×2^)
***f*_*max*_**	Maximum muscular tension (∈ ℝ^6^)
***M***	Inertia matrix (∈ ℝ^2×2^)
***C***	Coriolis force (*N*.*m* ∈ ℝ^2^)
τ	Segments torque (*N*.*m* ∈ ℝ^2^)
***B***	Damping term (*N*.*m* ∈ ℝ^2^)
***u***	Raw muscular activation (action) (∈ [0, 1]^6^)
κ	Multiplicative muscular noise (∈ ℝ)
ũ	Noisy muscular activation (∈ [0, 1]^6^)
q^*^	Target articular position (*rad* ∈ [0, 2π[^2^)
***q***	Current articular position (*rad* ∈ [0, 2π[^2^)
$\dot{\text{q}}$	Current articular speed (*rad*.*s*^−1^)
$\ddot{\text{q}}$	Current articular acceleration (*rad*.*s*^−2^)

## Author contributions

LP, OS, and JB contributed to conception/design of the work, and the acquisition, analysis and interpretation of data. LP, OS, and JB contributed to drafting/writing/revising the paper. LP, OS and JB gave the final approval of the version to be published and agreed to be accountable for all aspects of the work.

### Conflict of interest statement

The authors declare that the research was conducted in the absence of any commercial or financial relationships that could be construed as a potential conflict of interest.
