# Corrigendum: Unifying Speed-Accuracy Trade-Off and Cost-Benefit Trade-Off in Human Reaching Movements

**DOI:** 10.3389/fnhum.2018.00076

**Published:** 2018-02-20

**Authors:** Luka Peternel, Olivier Sigaud, Jan Babič

**Affiliations:** ^1^HRII Lab, Advanced Robotics, Istituto Italiano di Technologia, Genoa, Italy; ^2^Department for Automation, Biocybernetics and Robotics, Jožef Stefan Institute, Ljubljana, Slovenia; ^3^Sorbonne Universités, UPMC Univ Paris 06, CNRS UMR 7222, Institut des Systèmes Intelligents et de Robotique, Paris, France

**Keywords:** expected utility, hit dispersion, cost-benefit, speed-accuracy, arm reaching

There was a confusing notation of expectation in the Equations (2) and (4) in the original article. The original notation 𝔼(***s, u***)(…) could be misinterpreted as expectation of variables ***s*** and ***u*** themselves multiplied by the integral part inside (…), while in fact the notation represents the expectation of integral part inside the (…), where (***s, u***) only indicates that the random variables are ***s*** and ***u***. The clearer notation would be 𝔼_***s, u***_(…). The corrected versions of equations/text are below.

(2) $$J(u)=E_{s,u}\left(\int_{0}^{\infty }\left[e^{-t/\gamma }\rho R(s_{t})-\upsilon L(u_{t})\right]dt\right),\;$$

where 𝔼_***s, u***_(…) denotes the expectation over the random variables ***s*** and ***u***, which is the probability-weighted average of its argument over infinitely many repetitions.

(4) $$J(u)=E_{s,u}\left(\int_{0}^{\infty }\left[e^{-t/\gamma }\rho R(s_{t})-\upsilon L(u_{t})\right]dt+C\left(s_{f}\right)\right),\;$$

In the original article, we forgot to include the Acknowledgments section.

