# Corrigendum: Unifying Speed-Accuracy Trade-Off and Cost-Benefit Trade-Off in Human Reaching Movements

**DOI:** 10.3389/fnhum.2018.00143

**Published:** 2018-04-16

**Authors:** Luka Peternel, Olivier Sigaud, Jan Babič

**Affiliations:** ^1^HRII Lab, Advanced Robotics, Istituto Italiano di Technologia, Genoa, Italy; ^2^Department for Automation, Biocybernetics and Robotics, Jožef Stefan Institute, Ljubljana, Slovenia; ^3^Sorbonne Universités, UPMC Univ Paris 06, CNRS UMR 7222, Institut des Systémes Intelligents et de Robotique, Paris, France

**Keywords:** expected utility, hit dispersion, cost-benefit, speed-accuracy, arm reaching

There were several minor typos present in the Methods section of the original article.

In the paper we defined variables ***C*** and ***B*** as torque vectors, i.e., already multiplied by $\overset{\cdot }{q}$, and therefore they should not be multiplied by $\overset{\cdot }{q}$ in Equation (5). The corrected formulation of equation is:

(5) $$\ddot{q}=M{\left(q\right)}^{-1}(\tau -C(q,\dot{q})-g(q)-B(\dot{q}))$$

When reporting the model parameters in Table 4 in the paper, the values for inertia parameters *I*_1_ and *I*_2_ were accidentally switched with the values for center of mass distances *s*_1_ and *s*_2_. The corrected table is:

**Table:** 

*m*_1_	Arm mass (*kg*)	1.4
*m*_2_	Forearm mass (*kg*)	1.1
*l*_1_	Arm length (*m*)	0.3
*l*_2_	Forearm length (*m*)	0.35
*I*_1_	Arm inertia (*kg*.*m*^2^)	0.025
*I*_2_	Forearm inertia (*kg*.*m*^2^)	0.045
*s*_1_	Distance from the center of segment 1 to its center of mass (*m*)	0.11
*s*_2_	Distance from the center of segment 2 to its center of mass (*m*)	0.16

Since we previously defined operator × as element-wise multiplication, the joint torque calculation equation (page 12) should have a regular multiplication between matrix **f**_**max**_ and vector **ũ**, and not × operator. The corrected equation in the text is: $\tau ={\text{A}}^{⊤}\left({\text{f}}_{\text{max}}ũ\right)$.

The authors apologize for these typos and state that they do not change the scientific conclusions in any way.

The original article has been updated.

## Conflict of interest statement

The authors declare that the research was conducted in the absence of any commercial or financial relationships that could be construed as a potential conflict of interest.

