# Supplementary material for: Unifying Speed-Accuracy Trade-Off and Cost-Benefit Trade-Off in Human Reaching Movements
Source: Front Hum Neurosci. 2017 Dec 19;11:615. doi: 10.3389/fnhum.2017.00615 (PMC5770750; doi:10.3389/fnhum.2017.00615)
Supplement: Supplementary file 1 [file Presentation1.PDF]

Supplementary Material:
Unifying Speed-Accuracy Trade-off and Cost-Benefit Trade-off in Human Reaching Movements

1 MOVEMENT COST MAP

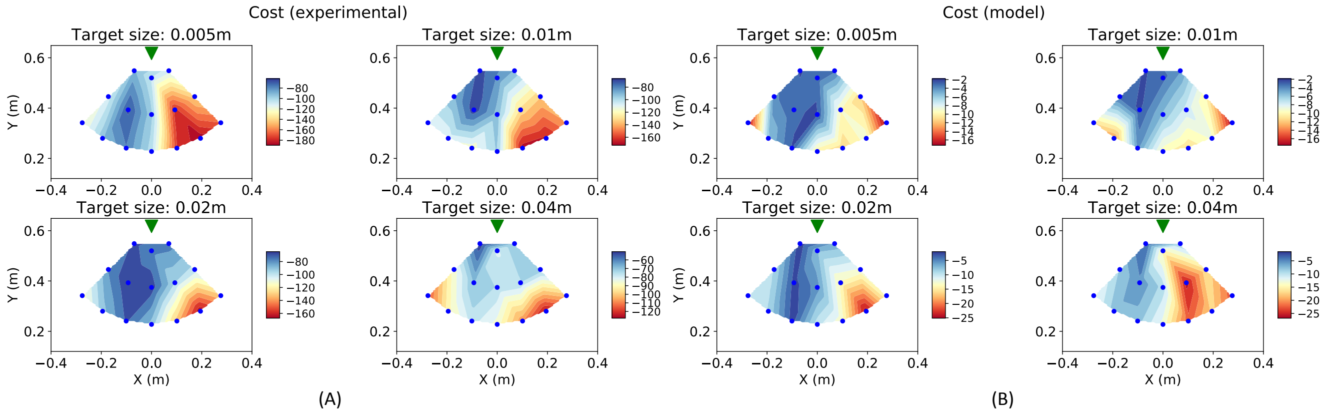

Figure S1: Cost map. (a): recorded from subjects. (b): obtained from the model. The color of a point in the reachable space illustrates the cost of a reaching movement from that point. The color-cost correspondence is given by the scale on the right hand-side. The scales of the costs cannot be compared between subjects and the model, as in subjects the cost estimation is obtained from EMGs, where the physical value of motor activations is lost.

The muscular cost of performing a movement from the different initial points described in Methods is shown in Fig. S1. The experimental cost of movement was calculated by using muscle activity measurements obtained from EMG. For each starting point, the muscle activity was integrated over the movement toward the target. The muscular activations necessary to compensate for the friction of the haptic manipulator at the end-effector were removed by estimating them through the arm model described in Methods and a model of the haptic manipulator friction.

One can see that some aspects are consistent between subjects and the model. First, the smaller the distance to the target, the lower the cost. Second, for large movements, the cost is consistently higher starting from the right than from the left side.

This latter point is explained by the fact that the optimal muscular strategy for performing these movements differs depending on the side. Actually, movements starting from the right are performed by moving simultaneously the elbow and the shoulder whereas when starting from the left, only the elbow is significantly involved, leading to a lower muscular effort. Another point is that the net cost is generally smaller for larger targets.

However, other aspects of Fig. S1 should be handled with care, as more local aspects are not robust neither in the experiments nor in simulation. From the experimental side, the study of individual EMG signals shows that muscular activations change a lot over subjects and over movements. From the simulation side,

the variability in the cost maps has two sources. First, estimating the cost at one point results from an approximation over a limited number of trajectories, thus it suffers from a significant variance. Second, the whole cost map is drawn out of 15 starting points only, thus variance at one point propagates over the whole surrounding area.

2 REMOVING THE PERPENDICULAR HIT COST FUNCTION

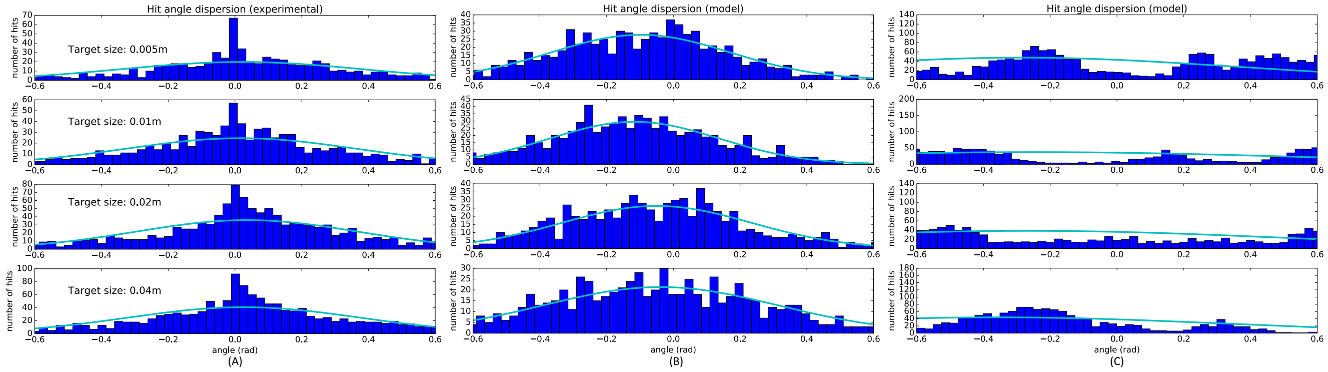

Figure S2: Distribution of hit angles obtained from 1500 trajectories starting from 15 points, for each target size. (a): recorded from subjects. (b): obtained from the model. (c): obtained from the model without the cost for not hitting the target perpendicularly. Dark blue histograms are obtained by counting all the trajectories that hit the target within a 0.025 radians range. Light blue Gaussians are fitted to the histograms.

As shown in Fig. S2A, subjects tend to hit the target perpendicularly. The computational model used in our simulation, described in main paper methods as Eq. (4), contains a specific cost term favoring trajectories that hit the target perpendicularly. This specific cost being not essential to our theory, we studied whether it could be removed from the model, with the hope that hitting the target perpendicularly just emerges from optimization, without this specific cost.

The hit angles of subjects and from the model with and without this cost term are displayed in Fig. S2C. As the figure shows, if we remove the perpendicularity cost function, the hit angles from the model become random, so the model without this cost does not explain hitting trajectories by itself. Furthermore, optimizing the controllers without this cost appeared much more difficult than with it, as this cost canalizes the trajectory search process into the right direction.

Actually, the hit angle of subjects still appears more peaked at perpendicular hit than those of the model with the perpendicularity cost function. This might be explained by a too small weight on the perpendicular hit cost function, resulting from the difficulty of performing meta-parameter search. Maybe increasing this weight would allow to a better match to the hit angle of subjects. Besides, we also considered including a manipulability criterion, but this did not improved the fit to experimental trajectories.
